# Progress in Fevers

**Published:** 1901-08-10

**Authors:** 


### PROGRESS IN FEVERS.

**Immunity.**—W. C. C. Pakes[1] deals with the question of immunity—natural and acquired. Natural immunity appears in certain *species*, *e.g.* man is susceptible to cholera and typhoid, a susceptibility unshared by the lower animals, but is insusceptible to swine plague, a common and virulent disease among swine. Pathogenic symptoms can, however, sometimes be produced by artificial inoculations in species naturally immune, *e.g.* the guinea-pig, which is naturally immune to diphtheria, dies of cardiac failure and paralysis after subcutaneous injection of a pure culture of the bacillus. Certain *races* also show special immunity, *e.g.* ordinary sheep and rats are very susceptible to anthrax; Angora sheep and white rats are insusceptible. There is also an *individual* immunity or susceptibility which undoubtedly exists albeit very difficult to explain. The history of any epidemic shows this. Only a few susceptible individuals get the disease in its mild early stage—more fall victims to its increased virulence, but a certain number of persons always escape. In considering infection the virus and the immunity of the individual must be taken into account. The latter may be put as a matter of dosage. Reduced to arithmetic, the requisite lethal dose or "immunity figure" is obtained by multiplying the strength of the virulent agent by the number of agents required to produce a fatal result. For example, if 20 pneumococci, the virulence of which is 20, are required to kill one rabbit, 400 pneumococci of which the virulence is 1 will be required to kill the same rabbit. This explains why diseases are endemic as well as epidemic. During the intervals between the epidemics when the virulence is low only a few highly susceptible people get a sufficient dose of the virus. As the virulence of the organisms increases by passage through the bodies of individuals, more people are able to get a sufficient dose, and the epidemic goes on increasing until the organism has reached its highest virulence. After that it declines, for all those susceptible to any ordinary dose of highest virulence have already succumbed to the disease. The relation between the dosage and the immunity figure will also explain differences in the incubation period.

Acquired immunity may be active or passive. Active immunity is obtained naturally by a previous attack. For many diseases this immunity, although not absolute, lasts many years, for others such as influenza, it is very short, the patient is left not more susceptible but *in statu quo.* Immunity can also be acquired by artificial injection with attenuated cultures. Repeated inoculations are necessary to confer a lengthened immunity. Immunity is also secured by injection of a sublethal dose of a virulent culture. It is not the organisms themselves, however, but the toxins they produce which form the chief lethal agent. These toxins may be described as intra- or extra-corpuscular—intra-corpuscular when present in the bodies of the bacteria as in typhoid bacilli, extra-corpuscular when formed in solution outside their bodies as in the case of tetanus and diphtheria bacilli. Immunisation by injection of these toxins is used as a prophylaxis in man. Vaccination means injection of an attenuated smallpox virus. Haffkine employs in India injection of an attenuated cholera virus as a prophylactic against cholera. Cultures of the dead organisms are employed in enteric fever and plague. Prophylactic injection of the extra-corpuscular tetanus or diphtheria toxins, except in minute insufficient dose, would be dangerous. The effect of the toxin on the organism is such that the tissues produce a neutralising substance. These self-evolved antitoxins are called "antikörpers" by the Germans. Wright suggests the name glutinins, for these bodies are closely allied to, if not identical with, the agglutinating agents. Passive immunity is obtained by injecting the serum of an animal already rendered actively immune. In active immunity the body produces its own antikörpers, or glutinins; in passive immunity they are injected ready made. Active immunity is better than passive as a prophylactic agent for the body takes time to produce its antikörpers; therefore the immunity is longer than in the passive form, which lasts only until the antikorpers introduced are eliminated. Passive immunity is of great value as a curative agent when the attempt to secure active immunity is probably useless or worse.

Sims Woodhead,[2] in discussing the subject, refers to the work of Metchnikoff, Meyers, Ehrlich, and many other observers. He inclines to the view that the production of antitoxin is the result of the special stimulation of certain special cells. The excessive amount of antitoxin produced suggests among other considerations that antitoxin formation proceeds long after the special specific stimulus has been removed.

**Vaccinia, Variola, and Varicella.**—Monckton Copeman[3] comes to the conclusion that the cowpox stated by Jenner to be derived from human smallpox must have been derived from the mild type of the disease obtained by inoculation in the human subject. Failing, in common with other observers, to produce cowpox from ordinary human smallpox, and unable to obtain material from inoculated cases, he passed the virus of ordinary variola through monkeys, which are very susceptible to the disease, and from them inoculated the calf. After one or two removes typical vaccinia was obtained in the calf, and children inoculated with the vaccine so obtained from the calf "took" in a typical and normal manner.

The same observer[4] has attempted to cultivate the supposed microbe of vaccinia and variola by employing a living medium. Collodion capsules of broth inoculated with pure glycerinated vaccine lymph were introduced into the peritoneal cavities of dogs and rabbits. Subsequent examination of unruptured capsules showed the presence of zoöglœa masses resembling groups of spores. The fluid contents of the capsules produced typical vaccinia in calves, while that of control capsules incubated at body temperatures outside the body produced no result.

Fünk[5] claims to have discovered a protozoon and to have established its claim as the causative agent in vaccinia. He has named it sporidium vaccinale in compliment to Pfeiffer, who described a sporozoon in 1887. The parasites, best seen in a fresh preparation, appear as clear vacuolated spaces in three forms. They are sufficiently large to be fished out with a platinum spatula and are accredited with the production of vaccinia in animals. The sporidium appears in the resulting pustules, and the animal is henceforth immune to vaccinia.

Kubin[6] lays stress on the importance of aseptic vaccination, particularly in the after-treatment of the pustules.

Montizambert[7] supplies notes of a particularly mild epidemic of smallpox invading Canada. One virulent intercurrent attack has occurred which was quickly stamped out. In the country it goes by the name of chicken-pox or German measles, or "cedar itch" in the lumber camps. Unvaccinated adults are the chief victims. F. Foord Caiger[8] indicates the distinctive points in the diagnosis of chicken-pox and smallpox. In the latter prodromal symptoms—rigor, vomiting, headache, and severe backache—occur and last for two days. In varicella prodromata are rare. If by chance they occur in an adult they only last one day as a rule. In smallpox the temperature falls as the rash appears, the converse holds good in chicken-pox. In the latter the rash is thickest on covered parts and it comes out in successive crops, whilst in variola the face, wrists, and ankles are favourite seats, and the rash comes out in only one crop. All papules which will appear do so before 36 hours have passed. The pock in varicella is more superficial, though it may be umbilicated and it matures in a couple of days. In variola the pock is more deeply situated, more indurated, and takes six or seven days to mature. The presence of chicken-pox scars is very important, for second attacks of this disease are very rare. A vesicular rash in a patient showing these is almost certainly not chicken-pox. On the other hand, the simultaneous presence of papules, vesicles, and pustules precludes smallpox. Chicken-pox has also to be diagnosed from a form of lichen apt to recur in young children. The papules rapidly become vesicular, show an urticarial base, and itch considerably. Doty[9] also lays stress in distinguishing variola from varicella on the character of the eruption, its method of appearance, and its distribution. In varicella the vesicles are very tender and easily torn, and are vesicular from the beginning, besides coming out in crops. The presence of shotty papules and vesicles on the hands and feet, particularly on the palms and soles, is very suggestive of variola.

[1] Clin. Jour., March 13. [2] Lancet, March 16, p. 783. [3] Brit. Med. Jour., May 11. [4] Med. Rec.. May 11, p. 735. [5] La Semaine Médicale, Feb. 20. [6] Med. Rec., April 6. [7] Brit. Med. Jour., May 11. [8] Clin. Jour., April 10. [9] Med. Rec , May 4.

